# Influence of sense of coherence on adolescents’ self-perceived dental aesthetics; a cross-sectional study

**DOI:** 10.1186/s12903-017-0405-2

**Published:** 2017-08-17

**Authors:** Aline Cavalcanti da Costa, Fabrícia Soares Rodrigues, Priscila Prosini da Fonte, Aronita Rosenblatt, Nicola Patricia Thérèse Innes, Mônica Vilela Heimer

**Affiliations:** 1 Dental School, University of Pernambuco, Camaragibe, PE Brazil; 2 School of Dentistry, University of Dundee, Park Place, Dundee, DD1 4HR UK

**Keywords:** Sense of coherence, Self-perception, Dental aesthetics, Adolescent

## Abstract

**Background:**

Sense of coherence (SOC) is a psychosocial factor capable of influencing perception of health, improving one’s ability to manage life. It is the central construct of salutogenesis. SOC allows for identification and mobilization of resources to effectively manage or solve problems, promoting health and quality of life. Using Wilson-Cleary’s conceptual model we hypothesized that SOC might contribute to self-perception of dental aesthetics. The aim of this study was to investigate whether SOC levels were related to self-perception of dental aesthetics against assessed normative orthodontic treatment need among adolescents.

**Methods:**

A cross-sectional study was conducted with 615 male and female adolescents aged 12 to 15 years. Data collection comprised socio-demographic and socio-economic characteristics, SOC (SOC 13), self-perceived dental aesthetics (Oral Aesthetic Subjective Impact Scale), and assessment of orthodontic treatment need (Dental Aesthetic Index). Statistical analysis involved Pearson’s chi-square test, Kruskal-Wallis test, Mann-Whitney test and multiple linear regression. Spearman’s correlation coefficient was calculated for the determination of the strength of correlations among the numerical variables. The level of significance was set at 5% (*p* < 0.05).

**Results:**

50.1% of the participants were classified as having a high SOC (≥ median). Overall, SOC was associated with self-perceived dental aesthetics (*p* = 0.048). In the adolescents with no orthodontic treatment need, those with a low SOC perceived their dental aesthetics more negatively than those with high levels of SOC. The multiple regression analysis demonstrated an inverse relationship between SOC and: 1) age (*p* = 0.007), SOC being higher in the younger age group; 2) self-perceived dental aesthetics (*p* = 0.001), a higher SOC being associated with those who had a positive dental self-perception.

**Conclusions:**

SOC was associated with self-perceived dental aesthetics and adolescents with a high SOC were more likely to perceive their dental aesthetics more positively. SOC did not seem to influence self-perception of dental aesthetics in adolescents who were clinically assessed as having an orthodontic treatment need, however, in those where there was no orthodontic treatment need, a low SOC was associated with a negative self-perception of dental appearance.

## Background

Current concepts of health include health/disease processes alongside the psychosocial aspects [1]. Oral health interventions that are based solely on clinical diagnoses and do not take into account patient perspectives and experiences, may not be fully effective [2]. In orthodontics, in addition to a clinical diagnosis, it is especially important to include subjective factors, because the impact of any malocclusion will be influenced by psychological state and personal and cultural values [3].

Several studies have shown the link between psychosocial factors and normative oral health/disease measures. In a long-term cohort study, in Cardiff, UK, self-esteem in adulthood was more strongly predicted (65% of the variance) by psychological variables such as: perception of quality of life; life satisfaction; self-efficacy; depression; social anxiety; emotional health and by self-perception of attractiveness. Only 8% of self-esteem was predicted by dental status [4]. A qualitative study of the effects of varying severities of developmental defects of enamel (DDE) with 10–15 years olds [5] found the presence of DDE to impact on individuals whose sense of self was defined by appearance and who depended on perceived approval from others about their appearance. Variations in the impact of DDE were related to defining aspects of sense of self rather than the enamel defects. Normative measures of malocclusion and treatment need have been shown to impact negatively on OHRQoL [6–9]. However, outwith the normative measures, self-perception of dental aesthetics seems to be the main factor that drives seeking orthodontic treatment [10–12] even where there is no clinically assessed (normative) treatment need. Individual’s perceptions of their need for orthodontic treatment are influenced by psychosocial factors including perceived norms of dental attractiveness [13].

Self-perceived dental physical attractiveness has wider effects and unattractive individuals may see themselves as less efficacious in social situations than their more attractive counterparts [13, 14]. Adolescents with malocclusions for whom treatment is highly desirable, and who perceive themselves in need of treatment, often suffer from low self-esteem, avoid smiling, report being the victims of bullying due to the appearance of their teeth and believe that having straight teeth increases popularity and improves success in life [15].

Antonovsky [1], an American sociologist, proposed the salutogenic theory that rethinks health away from the longstanding biomedical determinist philosophy of disease/health. The salutogenic model is presented as a counterpoint to the pathogenetic disease-associated model of health and is directly related to the promotion of health.

Sense of coherence (SOC) is the central construct of the salutogenic theory. It is a discrete attribute that protects the individual against the consequences of stress and helps to explain how some people have more ability to manage the adversities of life, identify and mobilize resources to resolve problems effectively and to promote health and quality of life [16, 17].

People with a high SOC perceive their health and quality of life to be good. They also have less fatigue, depression, loneliness and anxiety than those with a low SOC [1, 2]. Furthermore, the influence of SOC on OHRQoL has been tested in a school-based cluster randomized control trial investigated an intervention comprising seven sessions designed to improve child participation and feeling of empowerment [18]. They tested the intervention’s effect on SOC and, using the Wilson-Cleary model [19] theorized that it influenced the children’s OHRQoL. The intervention enhanced SOC and improved OHRQoL.

SOC can therefore be considered as a psychosocial factor capable of exerting an effect on how health is perceived. It has also been shown to be a predictor for establishing healthy behaviors and positive self-perceptions of oral health [20–22]. However, SOC has not been investigated in adolescents in relation to self-perception of malocclusion.

**Fig. 1:**
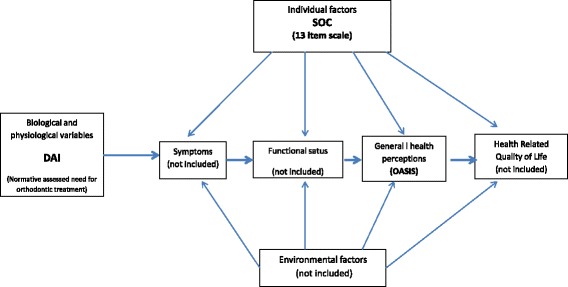
The Wilson-Cleary conceptual model of health-related quality of life [19]

**Fig. 2:**
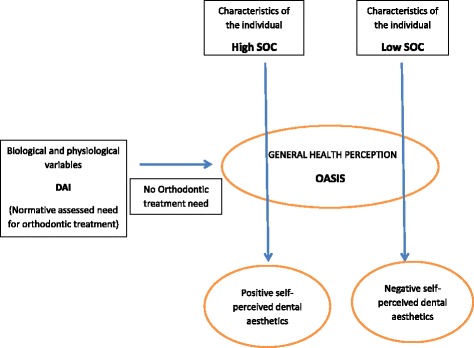
Proposed model of the relationship between SOC, Self-perceived dental aesthetics and normative orthodontic treatment need based on the Wilson-Cleary model [19]

The Wilson-Cleary conceptual model [19] (Fig. 1) provides a construct for thinking about how individual factors (such SOC), biological clinical variables (dental aesthetics), health perceptions and quality of life (QoL) are linked. Considering the strong effect that SOC can have on oral health related quality of life (OHRQoL) [2], we hypothetized that SOC might contribute to individuals’ perception of their aesthetics. Adapting the Wilson-Cleary model to demonstrate our hypothesis, we theorized that individual factors (in this case SOC), against the background of biological clinical variables (orthodontic treatment need), would influence oral health perception (self-perceived dental aesthetics). A low SOC, even the absence of normative orthodontic treatment need, would increase perceived orthodontic treatment need (Fig. 2). Ultimately, this may influence orthodontic seeking behavior (although we did not assess this).

Therefore, the aim of this study was to investigate whether SOC levels were related to self-perception of dental aesthetics against assessed normative orthodontic treatment need among adolescents.

## Methods

This study received approval from the Human Research Ethics Committee of the University of Pernambuco (Brazil) under process number 27434914300005207. All volunteers and their parents or guardians signed the consent form prior to data collection.

Data collection involved oral clinical examination of, and questionnaires completed by, adolescents.

### Participants

A cross-sectional study of adolescent girls and boys (12–15 years old), enrolled in the public school system in the city of Recife (a city with a metropolitan area population of 3.7 million in northeastern Brazil).

To ensure sample representativeness, simple random drawing was performed for each of the six administrative political regions, totaling 20 schools. In a second round of random selection, the subjects were chosen from the list of names from each school.

The sample size was calculated for the 13,750 students in this age range (from the Municipal Secretary of Education List), with an outcome prevalence of 0.50, a confidence interval of 95% and error margin of 5%. Taking into account a design effect equal to 1.5 and possible losses (20%), the sampled consisted of 674 students. Incomplete questionnaires were considered losses and represented 8.8% of the sample, resulting in a final sample of 615 students.

Those individuals currently or having undergone orthodontic treatment in the mixed dentition phase or with neuro-psychomotor impairment (as reported by teachers) that could affect completion of the assessment tools were excluded from the study.

### Measures

#### Sense of coherence

Sense of Coherence was measured using the short version of the Sense of Coherence questionnaire (SOC 13), validated for Brazilian adolescents [20]. This 13-item questionnaire has response options scored using a seven point Likert scale ranging from one (extremely negative) to seven (extremely positive). However, as values are inverted for items one, two, three, seven and 10, the values were corrected for this prior to total score determination (possible range; 13 to 91 points). Higher scores denote a stronger SOC. As there is no standardized cut-off point for categorizing high or low SOC, in common with standard methodology for handing SOC data, values above or equal to the median were considered as high SOC and below the median were considered as low SOC [23, 24].

#### Self-perceived dental aesthetics

The Oral Aesthetic Subjective Impact Scale (OASIS) [25] is composed of five questions addressing concerns and self-perception of dental appearance, and how dental irregularities negatively affect individual’s lives and their social relationships. Each question is scored on a one to seven Likert scale. The maximum score is 35 points, with higher scores denoting a more negative perception of dental aesthetics. The median was set as the cut-off point. The values above or equal to the median were considered negative self-perception, and the values below the median were considered positive self-perception [26].

#### Clinical measures

The Dental Aesthetic Index (DAI) [27], based on the World Health Organization criteria [28], was used to determine orthodontic treatment need. Clinical examinations were carried out in a reserved room at the school by a single orthodontist, twice with an interval of one week. The adolescent remained seated under natural light in front of the examiner. One calibrated examiner carried out clinical examinations and recorded malocclusion (DAI). The calibration process was performed prior to the survey in a group of 30 children aged 12 to 15 years old and, like in the main sample, written informed consent was obtained from the parents/legal guardians of these adolescents. Theoretical and clinical training and calibration exercises were arranged under the supervision of one benchmark examiner. The kappa value for intra-examiner reproducibility was 0.74. The DAI has 10 scored components, the total was submitted to an equation with weighting and the resulting DAI scores categorized as: Grade 1 (≤25 points) – little or no orthodontic treatment need; Grade 2 (26 to 30 points) – elective orthodontic treatment need; Grade 3 (31 to 35 points)-highly desirable orthodontic treatment need and Grade 4 (≥36 points) mandatory orthodontic treatment need. The scores were dichotomized as indicative of no orthodontic treatment need (Grade 1, ≤25 points) and indicative of need (Grade 2, 3, 4).

Since the eight components of the DAI require the anterior teeth for evaluation, individuals with loss of anterior teeth that prevented malocclusion evaluation were also excluded.

Socio-demographic characteristics were recorded using a previously designed chart and socioeconomic classification was determined based on the Brazilian Economic Classification Criteria [29]. These criteria include a series of questions related to the possession of household items and categorize families by socioeconomic class. In this study the families were classified as being in a high (classes A or B), intermediate (class C) or low socioeconomic class (classes D or E). Schooling of the household head was assessed by number of years of study and categorized as <8 or ≥8 years of study.

#### Analysis

Data analysis was performed using Statistical Package for Social Science (SPSS 21, IBM-United States), with a 5% margin of error. Either Pearson’s chi-square test was used to determine associations among the categorical variables. Odds ratios (OR) and respective 95% confidence intervals (CI) were calculated. The Kruskal-Wallis test was used for comparisons between categories of numerical variables, with multiple-comparison tests employed in cases of significant differences. Different letters in parenthesis denote statistically significant differences between corresponding analyses. The Mann–Whitney test was used to compare differences between two independents groups.

Spearman’s correlation coefficients were calculated to determine the strength of correlations among numerical variables, along with the Student’s t-test specific for the null hypothesis correlation. Adjusted multiple linear regression model determined the influence of SOC on the independent variables of age, sex and OASIS score. The dichotomized orthodontic treatment need scores were used as a reference score for SOC and OASIS.

## Results

Most of the sample was 12 (34.3%) and 13 years of age (33.5%), female (62.4%), belonged to families classified as intermediate socioeconomic class (71.2%) and head of the household schooling level was <8 years of the study for around half of the sample (50.4%).

After separating the sample into high and low SOC based on the median scores, it was approximately evenly divided between those with high SOC (50.1%) and low SOC (49.9%), 52.7% had a negative self-perception of their dental aesthetic. The DAI and OASIS scores demonstrated that 22.8% of the adolescents without orthodontic treatment needs had a negative self-perception of their dental aesthetics (Table 1).

**Table 1:** Descriptive statistics showing Sense of Coherence (SOC), self-perceived dental aesthetics (OASIS) and Orthodontic Treatment need (DAI) (*n* = 615)

Variable	n	%
Total	615	100.0
• Sense of coherence		
Low (< median)	307	49.9
High (≥ median)	308	50.1
• Self-perceived dental aesthetics (OASIS)		
Negative (≥ median)	324	52.7
Positive (< median)	291	47.3
• Orthodontic treatment need (DAI)		
No orthodontic treatment need (Grade 1, ≤25 points)	315	51.2
Assessed orthodontic treatment need (Grade 2, 3, 4)	300	48.8
• DAI + OASIS		
No orthodontic treatment need + negative self-perception	140	22.8
No Orthodontic treatment need + positive self-perception	175	28.5
Orthodontic treatment need + negative self-perception	184	29.9
Orthodontic treatment need + positive self-perception	116	18.9

A significant association was found between SOC and OASIS, as well as between SOC and DAI + OASIS. Without considering orthodontic treatment need, a high SOC was more prevalent among the adolescents with a positive self-perceived dental aesthetics compared to those who felt negatively (54.3% vs. 46.3%). When orthodontic treatment need was considered, for the adolescents who scored as having no orthodontic treatment need, those with a low SOC were more likely to have negative self-perceived dental aesthetics than those with a high SOC (60% vs. 43.4%) (*p* = 0.033) (Table 2).

**Table 2:** Sense of coherence according to OASIS as well as DAI + OASIS (*n* = 615)

	Sense of coherence							
Variable	High		Low		TOTAL		*p*-value	OR (95% CI)
	n	%	n	%	n	%		
Total group	308	50.1	307	49.9	615	100		
• OASIS								
Negative	150	46.3	174	53.7	324	100	*p* ^a^ = 0.048*	1.00
Positive	158	54.3	133	45.7	291	100		1.17 (1.00;1.37)
• DAI + OASIS								
No orthodontic treatment need + Negative self-perception	56	40.0	84	60.0	140	100	*p* ^a^ = 0.033*	1.00
No orthodontic treatment need + Positive self-perception	99	56.6	76	43.4	175	100		1.41 (1.11;1.80)
Orthodontic treatment need + Negative self-perception	94	51.1	90	48.9	184	100		1.28 (1.00;1.64)
Orthodontic treatment need + Positive self-perception	59	50.9	57	49.1	116	100		1.27 (0.97;1.67)

*significant association at 0.05 level

^a^Pearson’s chi-square test

No statistically significant associations were found between SOC and socio-demographic characteristics (Table 3).

**Table 3:** Sense of coherence according to socio-demographic characteristics (*n* = 615)

	Sense of coherence							
Variable	High		Low		TOTAL		*p*-value	OR (95 % CI)
	N	%	n	%	n	%		
Total group	308	50.1	307	49.9	615	100		
• Age (years)								
12–13	218	52.3	199	47.7	417	100	*p* ^a^ = 0.114	1.32 (0.94;1.85)
14–15	90	45.5	108	54.5	198	100		1.00
• Sex								
Male	127	55.0	104	45.0	231	100	*p* ^a^ = 0.060	1.17 (1.00;1.37)
Female	181	47.1	203	52.9	384	100		1.00
• Schooling of head of household								
<8 years	151	48.7	159	51.3	310	100	*p* ^a^ = 0.493	1.00
≥8 years	157	51.5	148	48.5	305	100		1.12 (0.81;1,53)
• Economic class								
High	59	54.1	50	45.9	109	100	*p* ^a^ = 0.527	1.19 (0.87;1.62)
Intermediate	218	49.8	220	50.2	438	100		1.09 (0.83;1.44)
Low	31	45.6	37	54.4	68	100		1.00

^a^Pearson’s chi-square test

Table 4 shows the findings for the associations between SOC and DAI + OASIS, age and sex. Mean SOC was significantly lower among adolescents with no treatment need + a negative self-perception compared to those with no treatment need + a positive self-perception. The findings showed a significant difference between adolescents between 12 and 13 years and those aged 14 and 15 years.

**Table 4:** SOC according to DAI + OASIS, age and sex (*n* = 615)

	Mean ± SD (median)
DAI + OASIS	
No orthodontic treatment need (DAI) + Negative self-perception	52.55 ± 9.28 (52.00)^(A)^
No orthodontic treatment need (DAI) + Positive self-perception	55.81 ± 10.20 (54.00)^(B)^
Need for treatment (DAI) + Negative self-perception	53.54 ± 10.80 (54.00)^(AB)^
Need for treatment (DAI) + Positive self-perception	54.68 ± 10.36 (54.00)^(AB)^
	*p* ^a^ = 0.045*
Age (years)	
12–13	55.06 ± 10.65 (54.00)
14–15	52.30 ± 9.17 (52.00)
*p*-value	*p* ^b^ = 0.006*
Sex	
Male	54.80 ± 10.23 (54.00)
Female	53.80 ± 10.28 (53.00)
*p*-value	*p* ^b^ = 0.104

Different letters in parenthesis (e.g. A, B and AB) denote statistically significant differences between corresponding analyses using Kruskal-Wallis

*significant difference at 0.05 level

^a^Kruskal-Wallis test

^b^Mann-Whitney test

There was a negative correlation between SOC with age and with OASIS (Table 5). SOC was higher among younger adolescents and those with a positive self-perception (lower OASIS scores).

**Table 5:** SOC correlated with age and self-perception scales (OASIS) (*n* = 615)

Variable	Spearman’s correlation coefficient
	r (s)
• Age	- 0.120 (0.003)^a^
• Self-perceived dental appearance (OASIS)	−0.130 (0.001)^a^

^a^significantly different from zero

Multiple linear regression with SOC as a function of age, sex, and OASIS show that age and OASIS score were inversely related to SOC, demonstrating that a higher SOC value was related to the younger adolescent age group and those with a lower OASIS (positive self-perception of dental aesthetic) score (Table 6).

**Table 6:** Multiple linear regression of SOC as function of age, sex and OASIS (*n* = 615)

Model	Coefficient		*p*-value
	Crude	Standardized	
Constant	73.300		<0.001^a^
Age	- 1.103	- 0.108	0.007^a^
Sex	0.903	0.043	0.284
OASIS	- 0.203	- 0.141	0.001^a^
R^2^ value	0.039		

^a^significant at 0.05 level

## Discussion

Just over half of this population scored as having high SOC agreeing with studies of individuals with the same age range [30, 31]. According to some researchers, SOC level stabilizes in mid-adolescence and can mediate stress in the same way as in adults [16, 32]. Indeed, adolescence is considered a crucial period for development of SOC, as adolescents face choices around identity, biopsychosocial changes and challenges in their development [33].

We found a negative correlation between SOC and self-perceived dental aesthetics (OASIS), as adolescents with a high SOC had lower OASIS scores and more positive self-perception of dental aesthetics. These results were confirmed by multiple comparison tests that verified significant differences in mean SOC between groups, as SOC was lower among the adolescents who had no treatment need and a negative self-perception of dental aesthetics. The importance of psychosocial factors as a health predictor should be noted, with individuals who have high SOC having positive health self-perceptions, better quality of life, better oral health behaviors, less fatigue, depression, loneliness and anxiety than to those with a low SOC [2, 16, 34].

The effect of an intervention to enhance SOC on oral health related quality of life in children was tested in an experimental study [18]. The intervention enhanced SOC and improved OHRQoL providing experimental evidence that SOC influences OHRQoL.

The relationship between sense of coherence and self-perceived dental aesthetics could help to explain why adolescents with no normative orthodontic treatment need perceive themselves to have a negative dental aesthetics. To understand adolescents who seek orthodontic treatment, when there appears to be no need, it is necessary to consider the individual as a whole – a biopsychosocial being. It is important to emphasize that self-perception of dental aesthetics is the main factor that drives seeking orthodontic treatment [10, 12] and this perception is influenced by psycho-social factors. However, orthodontic treatment is a specialized branch of oral health care and the access to such services is costly and remains limited in Brazil. Since people with a high SOC have a positive perception of their health and better quality of life [1, 2], strengthening the SOC in adolescents, may be a suitable avenue for promotion of satisfaction with self-perceived dental aesthetics [10], where there is a negative self-perception of dental aesthetic in spite of having no orthodontic treatment need.

Adolescents’ self-perceived dental aesthetics was evaluated using the OASIS. We found 47.3% of the adolescents to have a negative perception of their dental appearance, which may be explained by the high prevalence of treatment need identified through the DAI. Agreement between the OASIS and DAI has been previously reported [35].

Comparing normative treatment needs (DAI) and self-perception (OASIS), more than half of the adolescents perceived they had a treatment need, demonstrating their concern with their dental appearance. It is known that they compare themselves to their peers to decide on their standard for an acceptable smile [11, 14]. Previous studies have also compared normative and subjective treatment need between the perceptions of researchers and self-perceptions of adolescents [13, 36–38].

The association between age and SOC was non-significant in the bivariate analysis. However, mean rates of higher SOC were associated with younger participants in the multivariable analyses. According to Antonovsky [1], social, cultural and historical factors as well as one’s own life experiences contribute to the formation of SOC, either strengthening or weakening coherence, the process of which ends at around 30 years of age. In a study of adolescents with chronic diseases [39], girls were found to have significantly lower SOC than boys and to use less favorable coping mechanisms, which could exert a negative effect on SOC [13, 38]. Adolescent girls also experience higher degrees of interpersonal stress and are more sensitivity to stress than boys [40–42], although in this study, despite girls tending to have a lower SOC than boys, the difference was not a statistically significant.

Parents with a higher socioeconomic status tend to favor the development of initiative in their children, emphasize the negotiation of rules and are less prone to using cruel, punitive parental practices [43]. According to Antonovsky [1], such attitudes are fundamental to the development of SOC. However, there is conflicting evidence of the association between parental socioeconomic status and development of a high SOC in their children. One 30-year longitudinal study [44] found no correlation between parental socioeconomic status of parents and SOC in either their sons or daughters. Yet, in another study [44], the socioeconomic status can exert a strong influence on SOC during adolescence, but weak influence in adulthood. The authors suggest that other determinant factors affect SOC development, such as social support, social participation, cultural aspects, traditions and life experiences during child and adolescent development. Our study did not find socioeconomic or household head’s level of education to be significantly associated with SOC, possibly due to low variability in parents’ socioeconomic class. Neither did we find a link between level of education which would possibly have an indirect influence on SOC through socioeconomic status.

This study also aimed to evaluate the relationship between SOC and self-perceived dental aesthetics. It appears that, to date, studies have only addressed the relationship between SOC and self-perceived general health in participants with medical conditions such as epilepsy [45] and cerebral palsy [46]. To the best of our knowledge, there are no studies investigating the relationship between SOC and self-perceived dental appearance.

Most adolescents in this study with a positive self-perception of their dental aesthetics showed high SOC. Although novel, these findings lend support to the principles set forth by Antonovsky [1], which state that SOC is a global orientation that expresses a strong feeling of trust individuals have with regard to both internal and external environments. Among other factors, a high SOC has been shown to be associated with fewer symptoms stemming from oral problems, better perception of general health, better quality of life and higher self-esteem [2].

SOC was significantly associated with OASIS as well as DAI + OASIS. Adolescents with no treatment need, but who still had a negative self-perception of their dental aesthetics had a lower SOC. This suggests that SOC can influence on perceptions of dental appearance. Likewise, previous studies have found a significant association between self-esteem and perceptions of dental aesthetics [2, 15] individuals who saw themselves as less attractive had lower self-esteem than those who saw themselves as attractive. Self-esteem also influences self-perceived dental aesthetics [2]. A recent study of adolescents found that those with high self-esteem had less frequent impacts from their malocclusion and those who assessed the appearance of their teeth to be “poor” had worse oral health-related quality of life. The main finding further supported the premise in the Wilson-Cleary model [19] that factors concerning the individual, like self-esteem, have an important direct relationship between oral health-related quality of life and the opinions of young people concerning the appearance of their teeth [47].

This study used a cross-sectional design to verify the association among several variables at the same time with little or no additional cost. However, in interpreting the outcome of this type of study design, it is important to take into account its limitations. Due to the cross sectional design used, it is possible only to demonstrate associations and hypothesis directions of relationships based on the theory we have adopted. It is not possible to demonstrate causality. Longitudinal studies are needed to identify the direction and strength of the relationships identified. Another limitation was the use of the Dental Aesthetic Index (DAI) which does not represent all occlusal traits. It should be stressed, however, that the participants sampled for this research was representative of a population of 13,750 students between 12 and 15 years of age and validated instruments were used.

## Conclusions

SOC was associated with self-perception of dental aesthetics and adolescents with a high SOC were more likely to perceive their dental aesthetics more positively. Even where there was no clinically assessed orthodontic treatment need, adolescents with a low SOC had a negative self-perception of their dental appearance. Levels of SOC did not seem to influence self-perception of dental aesthetics in adolescents who were assessed as having an orthodontic treatment need. Younger participants had a higher SOC and more positive self-perception than older ones and no significant associations were found between SOC and socioeconomic status of the family, schooling of the head of the household or sex.

## Abbreviations

DAI: Dental Aesthetic Index
OASIS: Oral Aesthetic Subjective Impact Scale
OR: Odds ratio
SOC: Sense of coherence
